# *Dmp1* Promoter-Driven Diphtheria Toxin Receptor Transgene Expression Directs Unforeseen Effects in Multiple Tissues

**DOI:** 10.3390/ijms18010029

**Published:** 2016-12-26

**Authors:** Ahmed Al-Jazzar, Behzad Javaheri, Matt Prideaux, Alan Boyde, Cheryl L. Scudamore, Chahrazad Cherifi, Eric Hay, Mark Hopkinson, Michael Boyd, Martine Cohen-Solal, Colin Farquharson, Andrew A. Pitsillides

**Affiliations:** 1 Skeletal Biology Group, Comparative Biomedical Sciences, Royal Veterinary College, Royal College Street, London NW1 0TU, UK; aaljazzar@rvc.ac.uk (A.A.-J.); mhopkinson@rvc.ac.uk (M.H.); mboyd@rvc.ac.uk (M.B.); apitsillides@rvc.ac.uk (A.A.P.); 2 Orthopaedics & Trauma, University of Adelaide, Adelaide, SA 5005, Australia; matt.prideaux@adelaide.edu.au; 3 Dental Physical Sciences, Institute of Dentistry, Queen Mary University of London, Mile End Campus, London E1 4NS, UK; a.boyde@qmul.ac.uk; 4 Mary Lyon Centre, MRC Harwell, Science & Innovation Campus, Oxfordshire OX11 0RD, UK; c.scudamore@har.mrc.ac.uk; 5 Inserm U1132 & Université Sorbonne Paris Cité-Diderot, Rheumatology, Hôpital Lariboisière, Paris 75010, France; cherifichahrazad@gmail.com (C.C.); eric.hay@inserm.fr (E.H.); martine.cohen-solal@inserm.fr (M.C.-S.); 6 Roslin Institute, University of Edinburgh, Division of Developmental Biology, Easter Bush, Midlothian EH25 9RG, UK; colin.farquharson@roslin.ed.ac.uk

**Keywords:** osteocyte, diphtheria toxin receptor, bone

## Abstract

Mice harbouring a dentin matrix protein 1 (*Dmp1*) promoter-driven human diphtheria toxin (DT) receptor (*HDTR*) transgene (Tg) have recently been used to attain targeted ablation of osteocytes by diphtheria toxin (DT) treatment in order to define osteocyte function. Use of these Tg mice has asserted mechano- and novel paracrine regulatory osteocyte functions. To explore osteocyte roles fully, we sought to confirm the selectivity of DT effects in these transgenic mice. However, our findings revealed incomplete DT-induced osteocyte ablation, prevalent *HDTR* misexpression, as well as more prominent histopathological DT-induced changes in multiple organs in Tg than in wild-type (WT) littermate mice. Mechanistic evidence for DT action, via prominent regulation of phosphorylation status of elongation factor-2 (EF-2), was also found in many non-skeletal tissues in Tg mice; indicative of direct “off-target” DT action. Finally, very rapid deterioration in health and welfare status in response to DT treatment was observed in these Tg when compared to WT control mice. Together, these data lead us to conclude that alternative models for osteocyte ablation should be sought and caution be exercised when drawing conclusions from experiments using these Tg mice alone.

## 1. Introduction

Osteocytes, the cells trapped in mineralized bone matrix, are the most abundant cell type in the skeleton; comprising up to 95% of total bone cells. They originate from osteoblasts and during their osteoblast-to-osteocyte transition develop cytoplasmic processes that keep them connected to each other, to other osteoblasts, osteoclasts and to cells in marrow [1,2]. Osteocytes are very difficult to study because of their anatomical location within bone, and many of their proposed biological roles remain, therefore, incompletely defined and somewhat controversial. Their abundance, location and connectivity has meant that they have long been considered to serve a crucial sensory and regulatory role in mechanical adaptation and control of bone remodelling, and indirect evidence for their regulatory functions has grown [1,3,4,5,6,7,8,9].

The importance of osteocytes is also supported by their persistence within bone matrix, which indicates their role, at the very least, in regulating both initial osteoid mineralization and later bone mineral maturation phases [10,11]. Osteocytes are the primary source of sclerostin, the product of Sost gene, acting as an inhibitor of bone formation and in osteoblast regulation [12]. Recently, other provocative systemic functions beyond mechanotransduction have been proposed for osteocytes; it is thought that osteocytes regulate mineral homeostasis by secretion of fibroblast growth factor 23, which serves an endocrine role to regulate kidney phosphate reabsorption [1,11,13]. It has also been proposed that osteocytes control osteoclast-mediated resorption via their secretion osteoprotegerin and of the receptor activator of nuclear factor κB ligand (RANKL) [14,15,16,17].

This breadth and depth of proposed osteocyte roles has now been significantly extended in an intriguing set of studies using transgenic (Tg) (*Dmp1-HDTR*) mice. These purport to provide direct in vivo evidence for the physiological contribution of osteocytes to multiple skeletal and extra-skeletal functions. These mice express transgenic human diphtheria toxin receptor (the heparin-binding epidermal growth factor; *HDTR*) driven by a 10-kb dentin matrix protein (*Dmp1*) promoter fragment in order to facilitate selective osteocyte ablation upon diphtheria toxin (DT) injection (Tatsumi et al., 2007). Using this Tg mouse model, it has been reported that 70%–80% of osteocytes can be ablated, eight days after a single 50-µg/kg DT injection. Studies exploiting this reported ablation have been used to support roles in mechanotransduction and extensive additional systemic paracrine roles for osteocytes [18,19,20]. These include: (i) regulation of fat metabolism, through an influence of osteocytes upon the hypothalamus; (ii) modulation of primary lymphoid organs, through osteocyte-mediated support of B and T cell maturation via a maintenance of a lymphoid-specific stromal cell pool in bone marrow [20]; and (iii) control of mobilization from the hematopoietic stem cell niche (HSC) in bone marrow [19].

The 10-kb, as well as an 8-kb *Dmp1* promoter fragment [21,22] have also been used to overexpress genes of interest or Cre-recombinase for conditional deletion studies using the Cre-loxP system [8,17,23,24], and interpretation of these data is clearly totally reliant upon the selective targeting of osteocytes and the lack of any alternative direct actions in other tissue and organs. Use of Tg (*Dmp1*-*HDTR*) mice is additionally reliant on the attainment of a highly cell-specific ensuing *Dmp1*-driven DT sensitivity. This study examines more closely the reliance upon this highly osteocyte-selective targeting of the *Dmp1*-*HDTR* transgene and the specificity of the resultant sensitivity to DT upon which the use of these mice as a model for induction of osteocyte-less bone in vivo is based.

## 2. Results

### 2.1. DT Treatment Leads to Changes in Bone Formation Despite Inefficient Ablation of Osteocytes

**Figure 1:**
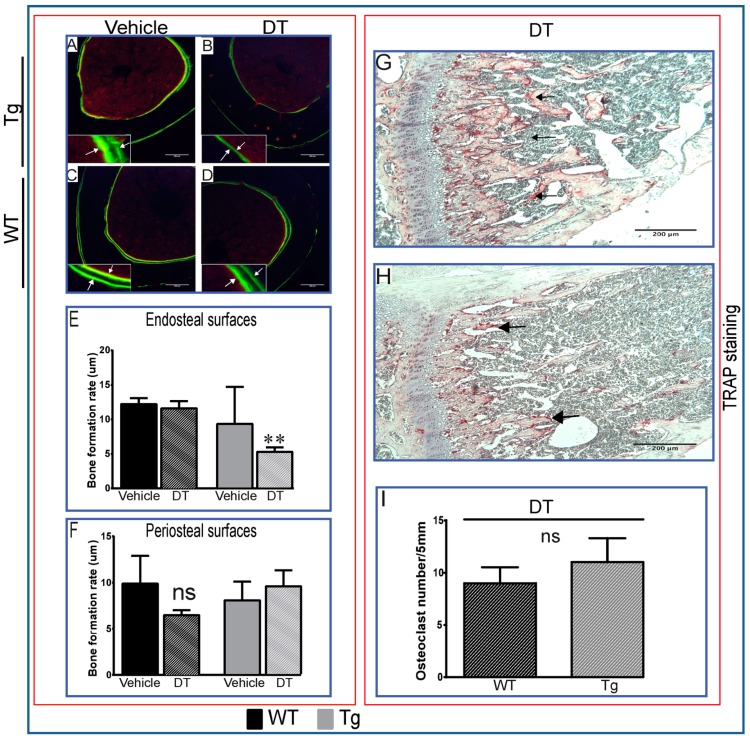

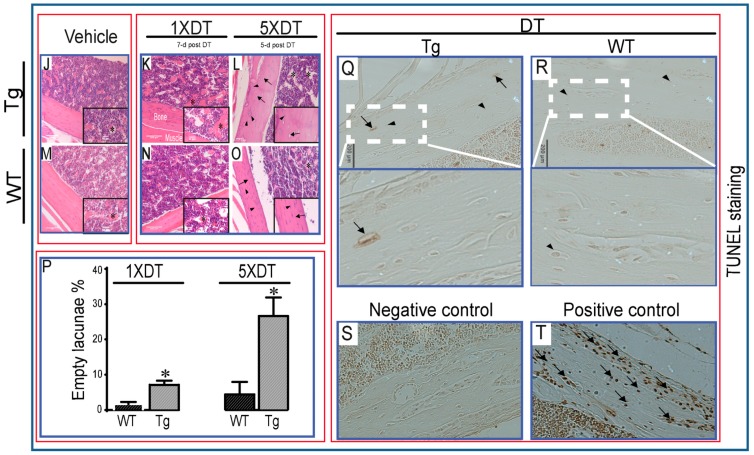
Diphtheria toxin (DT) injection leads to changes in bone formation despite inefficient ablation of osteocytes. Calcein double labelling (five-day interval) reveals robust levels of bone formation in vehicle-treated transgenic (Tg) (**A**) and WT mice (**C**) and diminished levels in only Tg (**B**), but not WT mice (**D**) five days after DT treatment; these changes were seen in the endosteal, but not the periosteal surfaces (**E**,**F**). White arrows indicate the inner and outer of two labels. Statistical comparisons: ** *p* < 0.05 vehicle and DT treated. TRAP staining for osteoclast activity for single DT-treated Tg (**G**; *n* = 3) and WT littermates (**H**; *n* = 3) (arrows indicate TRAP-positive osteoclasts) showed no significate differences between the two groups (**I**). Tg (**J**–**L** *n* = 4), but not WT (**M**–**O**; *n* = 4) mouse bones show marrow pathology, with marked congestion and distention of marrow sinusoidal blood vessels (*) at seven days after single DT (**K**) and more severe changes after five consecutive days of DT treatment (**L**); no comparable DT-induced changes in marrow composition were seen in WT mice (**N**,**O**). Significant osteocyte ablation (<30% empty lacunae; shown by arrowhead (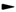), viable osteocyte; shown by arrow (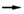), empty lacuna) was only observed in Tg mice treated with DT for five consecutive days (**M**) and only low levels (<10%) in WT and Tg mice after single DT treatment (**P**). Statistical comparisons: * *p* < 0.05 WT and Tg. Assessment of DT-induced apoptosis by TUNEL staining after single DT treatment in cortical bone of Tg (**Q**) and WT (**R**) revealed a very low number of apoptosis-positive osteocytes (arrow); arrowheads indicate negative cells. White-dotted boxes show a magnification of similar regions to better visualise the presence or lack of apoptotic cells. Negative (**S**) and positive (**T**) controls demonstrate a lack of staining in negative and many stained cells in positive control. Scale bar, 200 µm in the full overview images and 50 µm in the insets. Graphs represent the means ± SEM; ns = not significant.

Previous studies have described resistance to unloading-induced bone loss in Tg mice wherein significant levels of selective, DT-inducible osteocyte ablation were observed. Our initial studies sought to exploit this selective ablation to determine whether osteocytes also regulated physiological levels of cortical bone formation in freely-moving mice. Consistent with a regulatory role for osteocytes, we found that DT treatment also modified basal bone formation rate, as assessed by histomorphometry, with a significant reduction in bone accrual on the endosteal, but not the periosteal surface of the tibial midshaft in Tg mice (Figure 1A–F). Further examination of osteoclast activity by tartrate resistant acid phosphatase (TRAP) staining (Figure 1G–I) revealed that a single DT treatment did not significantly enhance TRAP activation in Tg (Figure 1G) compared to wild-type (WT) littermates (Figure 1H). Consistent with previous data [18], single DT treatment also produced changes in marrow composition in Tg (Figure 1J,K), but not WT littermates (Figure 1M,N), with marked DT-induced blood vessel dilation and significant increases in levels of sinusoidal congestion. Multiple, daily DT injections (five days) produced more marked sinusoidal dilation and more overt evidence of osteocyte ablation in Tg mice that was lacking in DT-treated WT mice (Figure 1L,P); it is noteworthy that these reduced levels of bone formation occurred over a time-course during which DT-induced histological marrow changes had occurred. Moreover, evaluation of DT-induced apoptosis by terminal deoxynucleotidyl transferase dUTP nick end labelling (TUNEL) revealed that osteocyte apoptosis was not significantly upregulated in Tg mice compared to WT littermates in response to DT treatment. Unfortunately, the number of TUNEL-positive cells was too low to quantify. In contrast to the bone tissue, many apoptotic cells were observed in the bone marrow of these mice (Figure 1Q–R). Furthermore, the strong labelling of cells in the positive control sections demonstrates the reliability of the TUNEL technique (Figure 1T). In addition, no TUNEL-positive cells were observed in the negative control sections (Figure 1S).

These data suggest that DT modifies bone-forming osteoblasts and/or their progenitor marrow recruitment in mice harbouring the Tg transgene and that this may be attributable to DT-induced ablation of osteocytes. However, in disagreement with this interpretation, we find that the numbers of ablated osteocytes were not extensive following a single DT injection in Tg and WT mice, with <10% dead osteocytes detected in Tg compared to <6% in WT littermates; more robust ablation was only attainable in Tg mice after multiple DT doses (<30% dead osteocytes or empty lacunae; Figure 1P). This was further confirmed by TUNEL staining, indicating that DT treatment does not lead to significant ablation of osteocytes as reported originally [18]. These data suggest that DT influences marrow composition and perhaps bone formation without the induction of significant osteocyte ablation, increasing osteoclast activity or the elevation of osteocyte apoptosis, but that DT-related modifications in bone formation are nonetheless reliant on *HDTR* expression driven via the *Dmp1* promoter.

### 2.2. Transgenic Mice Exhibit Generalized Adverse Health Status with DT Treatment

**Figure 2:**
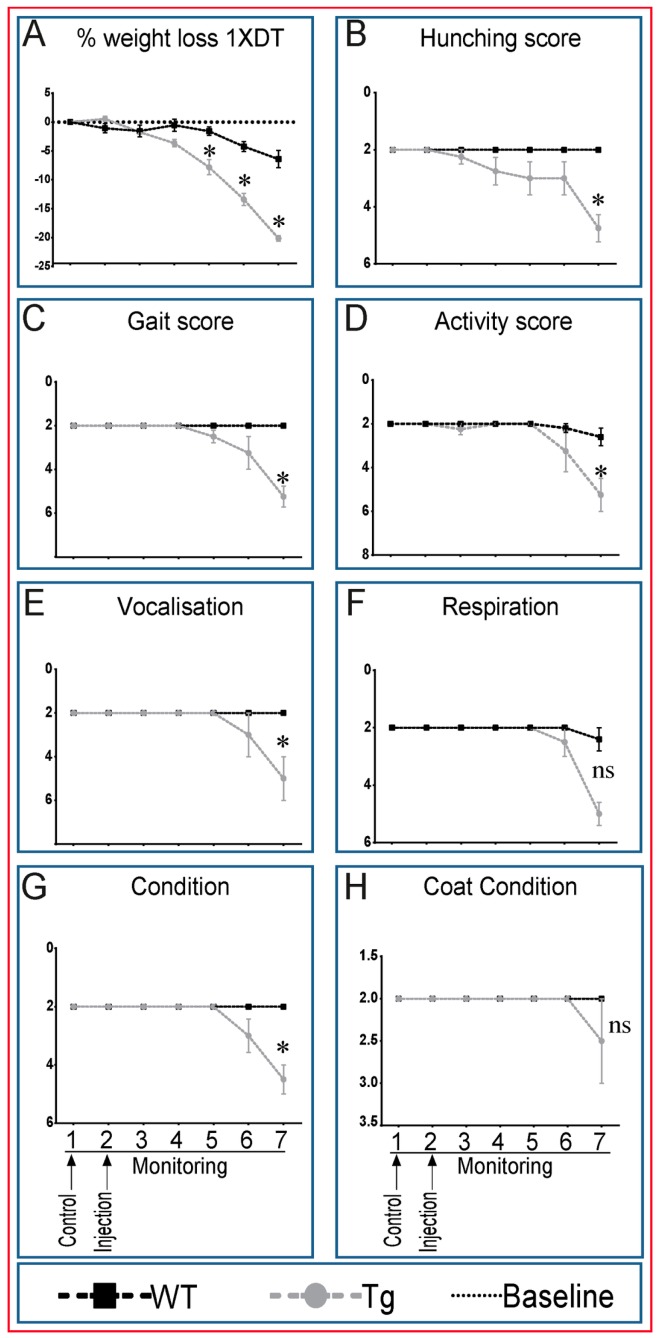
Transgenic mice exhibit generalized adverse health status with DT treatment. WT, but more markedly Tg mice showed substantial weight loss. This was apparent within two days after a single DT injection in Tg mice (**A**), which worsens to reach over 20% of starting body weight by Day 7. Tg mice also developed signs of distress and pain during the six-day period of DT treatment. These signs were manifested by a hunched back as a sign of distress (**B**). The change in gait, which might be due to pain (**C**), reduced activity (**D**), loud vocalisation (**E**), increased respiratory rate (**F**), deteriorated body condition (**G**) and deterioration of coat condition (**H**), which suggests lack of grooming and personal care. These signs suggest that DT impacts severely on the welfare particularly of treated Tg mice. Graphs represent the means ± SEM. Statistical comparisons: * denotes *p* < 0.05 between DT-treated WT and Tg mice; ns = not significant

The rapid onset of these DT-induced bone marrow changes without marked osteocyte ablation led us to explore whether Tg mice showed generalised signs of ill-health post-DT injection. We undertook formal evaluation of mouse welfare status by measuring several well-known health indicators after single DT treatment [25,26] (Guide for the Care and Use of Laboratory Animals, National Research Council, 2010). This indicates that Tg mice, not WT littermates, show clear symptoms consistent with significant ill-health, as early as three days after DT treatment, with a hunched back, bulbous nose, lack of appetite, pain vocalization behaviour and loss of weight (Figure 2A–H). The severity of these signs worsened by Day 7 post-DT, at which point mice had lost more than 20% of their original body weight, and the experiment, under UK Home Office regulations, had to be terminated (Figure 2A). These signs indicate dramatic DT effects on health status and suggest that deterioration is due to targeting of multiple organs in Tg mice.

### 2.3. Multiple Tissues Show Misexpression of Dmp1-Driven HDTR mRNA and Broad Expression of Dmp1

**Figure 3:**
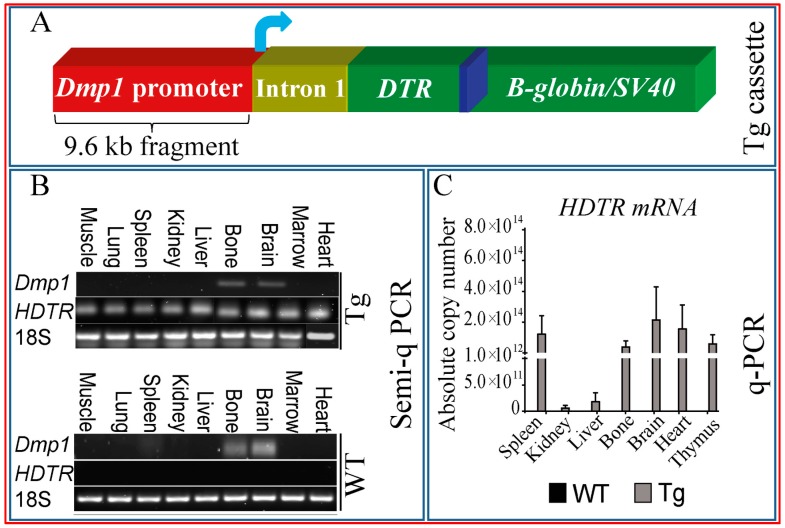

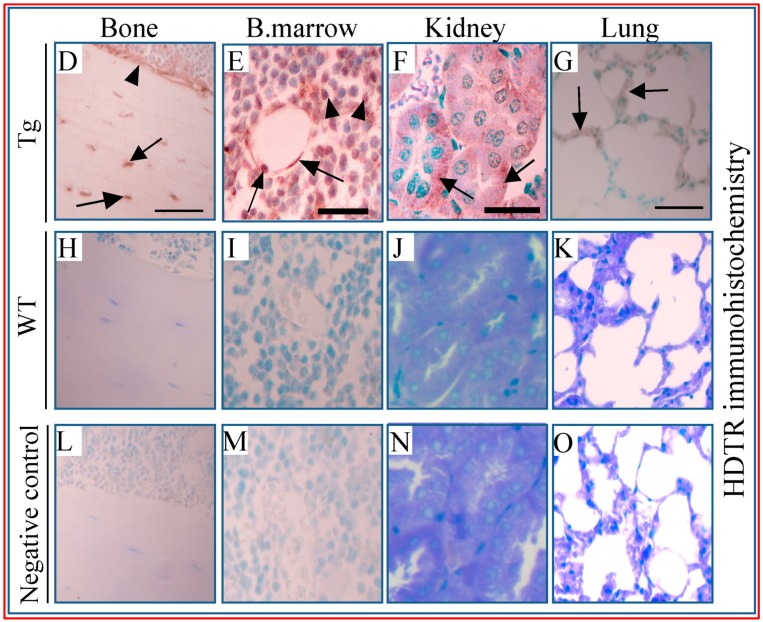
Multiple tissues show the misexpression of *Dmp1*-driven *HDTR* mRNA and broad expression of *Dmp1*. (**A**) A 9.6-kb transgenic cassette (50-flanking region, exon 1, intron 1 and part of exon 2 of the mouse *Dmp1* gene) was fused to human DTR cDNA using a toxin receptor-mediated cell knockout system and injected into fertilized egg pronucleus. Blue arrow indicates direction of transcription [18,28]. Semi-quantitative mRNA analysis reveals *Dmp1* mRNA expression in only bone and brain and a lack of selective *HDTR* mRNA expression, with the distribution in almost all tissues examined from Tg, but not in WT mice (**B**), respectively. Quantitative PCR of *HDTR* mRNA transgene expression analysis revealed that *HDTR* has a broad expression in multiple tissues (**C**). Samples were normalised to GAPDH. Immunohistochemical staining against HDTR protein in selected tissues revealed the expression of HDTR protein in osteocytes (**D**, arrow; 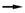) as wells as osteoblasts (**D**, arrowhead; 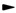), bone marrow cells (**E**, arrowhead) and around vessels (**E**, arrow), kidney tubules (**F**, arrow), and lung alveolar wall (**G**, arrow). Respective tissues show negative staining in WT (**H**–**K**). (**L**–**O**) are PBS negative controls. Scale bar, 200 µm.

To explore the specificity of transgenic 10-kb *Dmp1-driven* HDTR (Figure 3A: transgenic construct) expression in Tg mice, we evaluated *HDTR* mRNA, as well as protein expression in multiple WT and Tg mouse tissues. Semi-quantitative PCR showed that the *HDTR* transgene was expressed in a wide range of tissues, such as liver, kidney, spleen, heart, marrow, lung, as well as bone in Tg, but not WT littermates (Figure 3C). This pattern of *HDTR* mRNA expression did not, however, match accordingly with endogenous *Dmp1* mRNA expression patterns, which were restricted to only brain and bone tissues in Tg and WT mice, suggesting *Dmp1*-independent *HDTR* transgene expression mechanisms in multiple tissues. Both *HDTR* and *Dmp1* PCR products were validated by sequencing and restriction digestion [27].

We also further investigated the expression level of *HDTR* by quantitative PCR (qPCR) to determine whether these correlate with the histopathological impact of DT treatment. This qPCR analysis confirmed that *HDTR* showed a wide tissue distribution with the highest levels of mRNA expression in brain, somewhat less so in heart, spleen, thymus and bone and very low levels in kidney and liver (Figure 3C).

Further examination of HDTR protein expression was sought using immunohistochemical labelling for HDTR in selected tissues. Osteocytes and osteoblasts in bone (Figure 3D), as well as bone marrow and sinusoidal blood vessel cells (Figure 3E), kidney tubules (Figure 3F) and lung alveolar wall (Figure 3G) were all labelled positively for HDTR in Tg mice, but not WT littermates (Figure 3H–K). This suggests that cells in multiple tissues express HDTR and that the deleterious effects of DT might not be limited to bone or to tissues expressing endogenous *Dmp1*.

### 2.4. Pathological Changes Are Evident in Multiple Tissues Following DT Injection

**Figure 4:**
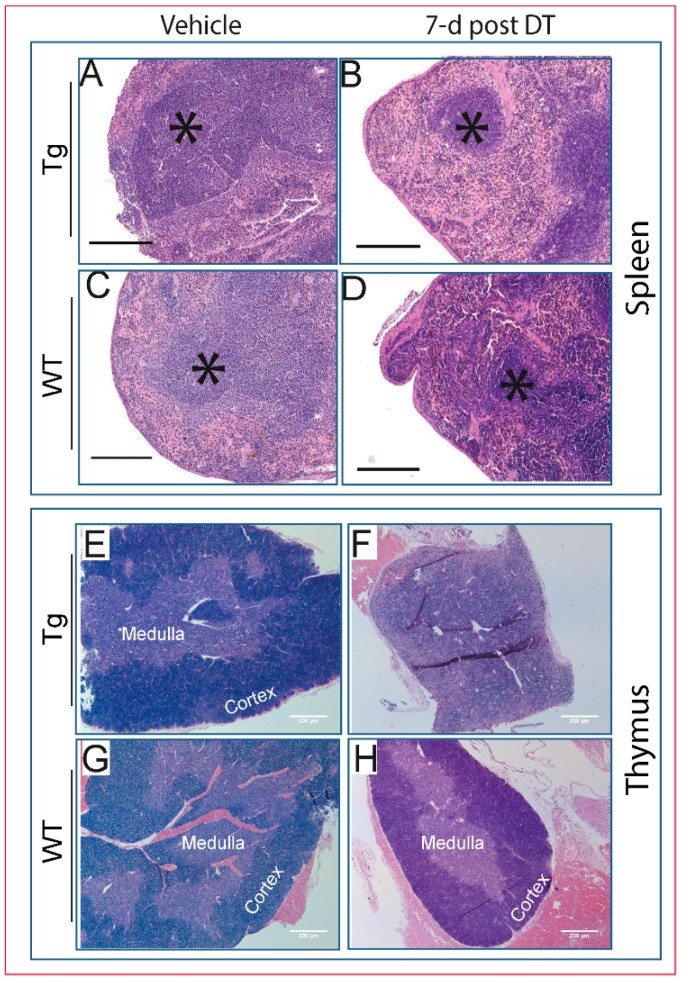
DT induces severe atrophy in primary lymphoid organs. H & E staining of spleens from Tg (**A**,**B**) and WT (**C**,**D**) mice shows atrophy and diminished white pulp (*) seven days after single DT treatment in Tg (**B**), but less so in WT (**D**) mice; scale bar 250 µm. (**E**,**F**) Tg exhibit more severe thymic atrophy than WT (**G**,**H**) mice seven days post-DT. Scale bar 200 µm.

Based on ill-health post-DT and *HDTR* misexpression patterns, we examined whether DT administration produced pathological changes in non-skeletal tissues, likely independently of osteocyte function and endogenous *Dmp1* promoter activity in Tg mice. Histopathological assessment revealed that DT-induced changes included more severe lymphoid atrophy in the spleen (Figure 4A–D) and thymus (Figure 4E,F) in Tg than WT mice (Figure 4G,H).

**Figure 5:**
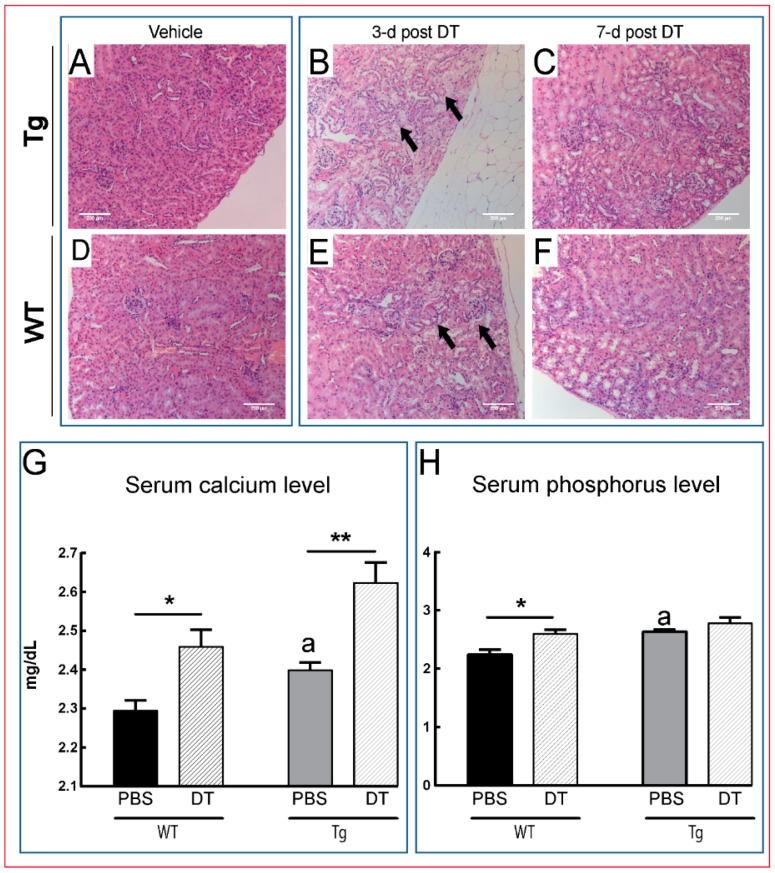
DT induces severe kidney damage. Tg (**A**–**C**) and WT (**D**–**F**) mice both show overt tubular necrosis (arrows; 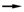) as early as three days after DT treatment (**B**,**E**), before any overt osteocyte death (Figure 1J–P). Kidney shows an apparent recovery in these pathological changes seven days post DT (**C**,**F**) with increased serum calcium and phosphorous levels in treated Tg mice seven days post DT (**G**,**H**). Scale bar 200 µm. Graphs represent means ± SEM. Statistical comparisons: * *p* < 0.05 and ** *p* < 0.01 vehicle and DT treated within each genotype; (a) denotes significant differences between vehicle-treated WT and Tg mice (*p* < 0.05).

Acute tubular necrosis was seen in the kidney in Tg mice by three days of DT treatment; similar, but less severe changes were observed in DT-treated WT littermates (Figure 5A,B,D,E). The kidney lesions appeared to exhibit rapid reversibility with regenerative tubules evident seven days after a single DT treatment (Figure 5C,F). Despite an absence of very high osteocyte ablation levels, we nonetheless observed significant increases in serum calcium in DT-treated Tg and WT mice (Figure 5G; *p ≤* 0.01 and *p ≤* 0.05, respectively). Furthermore, phosphorus levels were increased in both Tg and WT mice in response to DT treatment, but failed to reach statistical significance in Tg mice compared with vehicle-treated mice (Figure 5H; *p ≤* 0.05). Moreover, it was noted that basal circulating levels of both calcium and phosphorous were higher in Tg compared to WT littermate mice, which could be attributed to reduced renal function (Figure 5G–H; *p ≤* 0.05). Together, these data indicate that DT treatment produces deleterious effects in multiple tissues in both Tg and WT littermates, suggesting that any contribution of osteocyte ablation is likely complicated in these mice by such “off-target” changes. In addition to kidney and lymphoid tissues, we also evaluated histopathological changes in liver, muscle, brain and lung, all of which appeared histologically normal in both WT and Tg mice.

### 2.5. Dephosphorylation of EF-2 Reveals Direct DT Toxicity in Multiple Tissues in Tg Mice

To examine whether pathological changes in extra-skeletal tissues are due to direct DT toxicity, we exploited its known mechanism of action to track its cellular effects. DT, which separates into two fragments, containing a catalytic domain (21.1 kDa) and the receptor and transmembrane domain (41.2 kDa) [29,30,31], binds to cell surface DTR, causing membrane internalization and the formation of lysosomal vesicles in which the DT transmembrane domain forms a pore. This pore permits translocation of the catalytic DT domain into the cytosol [31,32] where it promotes NAD^+^-dependent ADP ribosylation of elongation factor-2 (EF-2), which leads to inhibition of protein synthesis and apoptosis [31,33]. Accordingly, dephosphorylation of EF-2 is used to identify tissues in which DT is effectively translocated into the nucleus, and reduced levels of phosphorylated EF-2 (pEF-2) in tissue can also be monitored to signpost direct effects of DT toxicity.

**Figure 6:**
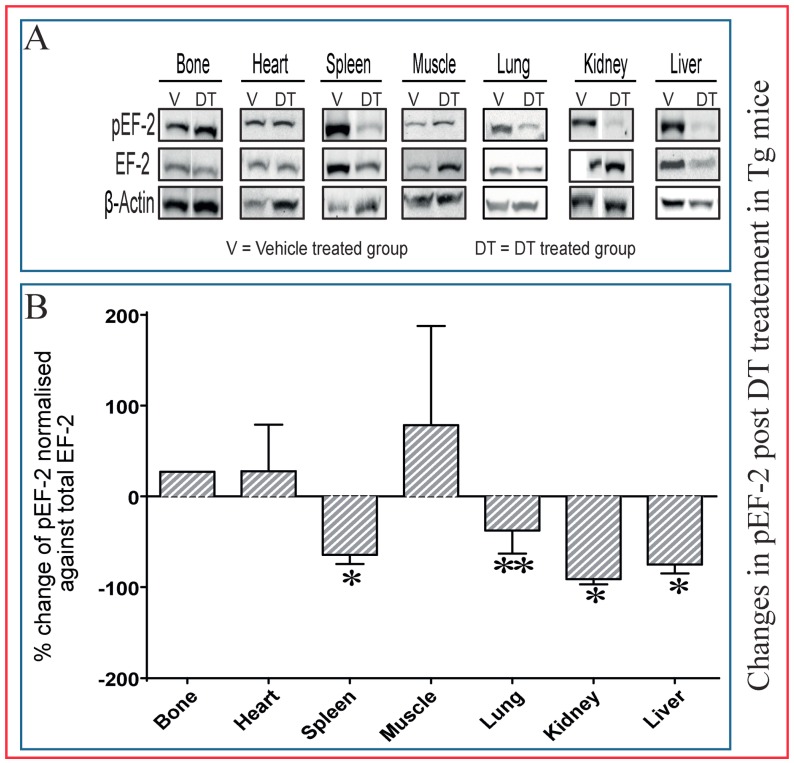
Dephosphorylation of EF-2 reveals direct DT toxicity in multiple tissues in Tg mice. Bones of DT-treated Tg and WT mice show equivalent pEF-2 levels to control mice seven days post DT treatment (**A**: blots; **B**: quantification), which suggest minimal effects in bone. In kidney, spleen, lung and liver, there is a significantly lower pEF-2 level indicative of direct targeting of these tissues by DT. In addition, muscle and heart also show non-significant changes in pEF-2 levels compared to control mice seven days post treatment. (**A**) Representative image of one out of four; (**B**) quantification of all groups, *n* = 4. * *p* < 0.05 and ** *p* < 0.01 compared with vehicle treated Tg mice.

Examination of pEF-2 (and total EF-2) levels in diverse tissues by immunoblotting showed that DT treatment of Tg leads to reduced pEF-2 levels (EF-2 inactivation) in the histologically-affected tissues, such as spleen and kidney; thus indicating that DT dephosphorylates EF-2, seven days post-treatment, to directly inactivate EF-2 in vivo in a wide range of tissues in these Tg mice (Figure 6). DT-induced declines in measured pEF-2 levels were not observed in bone samples (Figure 6A,B). The reduction of pEF-2 after DT treatment in Tg mice suggests that multiple tissues are directly targeted by DT, likely independently of osteocytes.

## 3. Discussion

Our data provide a clear demonstration of inefficient osteocyte ablation and *HDTR* misexpression, EF-2 inactivation and tissue pathology in several organs, as well as clear ill-health within days of DT treatment in mice with a 10-kb *Dmp1*-driven *HDTR* transgene. These data indicate that conclusions regarding osteocyte function should be viewed with prudence when based solely on this model. They question the use of these Tg mice as a reliable model for DT-inducible, specific osteocyte ablation, as they show direct downstream DTR-mediated EF-2 inactivation in multiple tissues.

A single DT dose (50 µg/kg), as used by Tatsumi et al. (2007), was reported to generate >80% of osteocyte death [18]. In contrast, we found this regimen to produce only minimal levels of osteocyte ablation. Our attempt to emulate the high osteocyte ablation levels necessitated the deployment of multiple DT doses. This, however, only highlighted the clear signs of illness, discomfort and pain within 48 hours of administration in Tg mice. Compliance with UK Home Office welfare guidelines required that we culled DT-dosed Tg mice by Day 7 when dosed once and by Day 3, when dosed daily. The main criterion for culling was a greater than 20% loss in body weight, with additional lowering of body temperature, staggered gait, hunching, loss of condition and vocalization. This severely limits the scope to use this model and likely complicates conclusions drawn concerning the physiological role of osteocytes both in regulating bone and non-skeletal tissues.

Further studies with these Tg mice have shown that osteocyte networks are disrupted by 20 [20] and 50 µg/kg DT [19]. These studies have attributed system-wide endocrine roles to osteocytes. Although our data confirm some aspects of these previous findings, we nonetheless find complex extra-skeletal changes, unlikely attributable to any selective effect of DT upon osteocyte survival. An alternative view is that osteocytes serve even more widespread and acute roles in maintaining health status and that these are manifest despite the low levels of osteocyte ablation we report herein. For example, we report that DT-induced marrow changes resembled closely those changes observed previously in Tg mice [20]. Despite this, our studies suggest that these widespread changes are instead, direct, off-target DT effects. Indeed, bone marrow in Tg mice is positive for *HDTR* expression in the absence of *Dmp1* expression. This may explain the sensitivity to DT-induced changes in marrow composition, which may, in turn, underpin later disruption of bone formation on the endosteal surface. Absence of any DT-induced modification in periosteal bone formation is perhaps consistent with this possibility. The observed dilatation of sinusoids and lowering of bone formation by DT treatment in Tg mice, but not WT littermates, might also be consistent with DT-mediated targeting of local stromal cell and osteoblast progenitors. 

Our methods show few apoptotic osteocytes in DT-treated Tg mice (Figure 1Q), but none in the bones of WT mice injected with DT (Figure 1R), making quantification or statistical analysis of these data unwarranted. The reliability of our TUNEL technique is nonetheless demonstrated by strong labelling in almost all cells following DNAase I-treatment and by the lack of labelling in negative control sections incubated in the absence of transferase (Figure 1S,T). In addition, our findings indicate that both spleen and kidney in Tg mice are also positive for *HDTR* and show DT-related decreases in pEF-2 levels. Cross-tissue gene expression analysis of the *HDTR* transgene reveals misexpression in Tg, but not WT mice in a wide range of tissues. This is consistent with the extent of pathology in the affected tissues. Furthermore, whilst mice are naturally more resistant to DT than humans, it remains possible that the principle of using DT treatment to target only transgenically-expressed DTR in mouse models is perhaps questionable. We consider that the investigations into the endogenous sensitivity of WT mouse tissues to DT are beyond the scope of our present study. In a study investigating the effect of DT lethal dose on resistant mice, the authors reported that mice do not exhibit the same impaired cardiac function as humans and guinea pigs in response to DT administration. This study also found, however, that in mice that had received 2000× the minimum lethal dose for guinea pigs (0.06 μg per 250 g) that kidney and skeletal muscle were the most affected tissues; interestingly, with normal function in cardiac muscle suggesting different tissue sensitivity to DT [34]. This observation was also applicable to the pEF-2 reduction level where tissues like spleen, kidney and liver showed direct evidence of DT toxicity by exhibiting a reduction of pEF-2 following DT treatment. However, lung showed a significant reduction of pEF-2 without evidence of histopathology, which highlights the difference of tissue sensitivities. Clearly, these effects might be highly dependent on species, but it nonetheless appears to suggest that some tissues are naturally more sensitive to DT than others. On this basis, we conclude that pathological changes in these tissues are likely due to direct action of DT rather than an indirect consequence of osteocyte ablation.

The value of these Tg mice and any DT-based induction of cell death using promoter-based transgene delivery is contingent upon both the specificity of transgene expression in the target tissue and the lack of any DT effects in a non-transgene-dependent manner. The extra-skeletal effects of DT in these Tg mice may arise due to several factors, including the non-specificity of the 10-kb *Dmp1* promoter fragment and/or misexpression of DTR in non-targeted tissues. Herein, we find that endogenous *Dmp1* expression is primarily detected in bone and brain. Indeed, previous studies reported that *Dmp1* is expressed primarily in bone, but also in submandibular salivary gland, kidney and brain [35,36,37,38]. The *HDTR* transgene is, however, detected in multiple tissues. This agrees with previous studies reporting the misexpression of DTR in similar DT-based transgenic models [39,40,41]. Furthermore, Jung et al. (2002) and Probst et al. (2005) described the limitations of *HDTR*-Tg mice, including promiscuous DTR expression, which causes mouse death after repeated DT injection [42,43]. The multitude of changes including histopathological effects in several of these and other organs in DT-treated Tg mice, including spleen, kidney, thymus and bone marrow, is therefore more likely due to wide and non-targeted expression of *HDTR*.

We also observed higher basal levels of both serum calcium and phosphorous in Tg compared with WT mice, which may be highly illuminating. These data suggest that *Dmp1* promoter-driven expression of transgenic *HDTR* (also known as heparin-binding epidermal growth factor (HB-EGF)), under untreated basal conditions, creates aberrant calcium and phosphate metabolism by interfering with endogenous homeostasis in either the skeleton and/or likely major regulating of the parathyroid gland, kidney and gastrointestinal (GI) systems. We speculate that transgenic overexpression of HB-EGF (*HDTR*) is sufficient to modify circulating calcium and phosphorous levels. This is consistent with the neutralization of de novo paracrine activation of glomerular epithelial cells by HB-EGF or EGF receptor antagonists to limit renal failure in immune-mediated vasculitis [44]. It is also in agreement with the role of juxtacrine HB-EGF receptor activation by membrane-anchored HB-EGF in regulating transepithelial renal resistance [45] and with the identification of HB-EGF receptor pathway inhibition as a potential target for anti-hypercalcaemic therapy [46]. Furthermore, Yano et al. (2004) found that calcium-sensing receptor-mediated HB-EGF receptor transactivation results in increased PTHrP secretion in PC-3 human prostate cancer cells, blockable by prior incubation with an antihuman HB-EGF antibody [47]. Together, these data show roles for the HB-EGF receptor (*HDTR*) in calcium homeostasis and suggest that its transgenic overexpression, without DT treatment, is sufficient with activation via alternative ligands to dysregulate basal serum calcium/phosphate levels. Future studies that we consider to be beyond the scope of this present study may, for example, measure 1,25-Dihydroxyvitamin D3 (VitD3), parathyroid hormone (PTH) and calcitonin levels to help decipher these mechanisms.

## 4. Materials and Methods

### 4.1. Animal Model

The Tg mouse model expressing *HDTR* driven by the 10-kb *Dmp1* promoter was generated by Tatsumi et al., as described previously [18]. Eight-week-old Tg mice were purchased from Riken Japan. To distinguish between Tg and WT littermates, genotyping was carried out on ear biopsies using direct PCR lysis reagent (Viagen Biotech Inc., Los Angeles, CA, USA), and DNA was analysed by strand PCR. Mice were raised under standard laboratory conditions and experiments were conducted in compliance with the ARRIVE (Animal Research: Reporting of *In Vivo* Experiments) guidelines for reporting. Briefly, mice were housed up to 4 per cage in polypropylene cages with wood chip and paper bedding and provided standard mouse chow and water ad libitum throughout the study. Weaners up to 8 weeks of age were fed a standard rodent breeding diet and thereafter a standard rodent maintenance diet (Special Diet Services, South Witham, UK). All procedures complied with and were undertaken under the Animals Scientific Procedures Act by authorisation of a Project Licence granted by the UK Home Office (Awarded on 24 April 2014) with the approval of the Royal Veterinary College’s Ethics and Welfare Committee (London, UK). 

### 4.2. Diphtheria Toxin Administration

Diphtheria toxin (Sigma; Poole, Dorset, UK) was dissolved in sterile phosphate-buffered saline (PBS, used in vehicle-treated mice) and used to treat both Tg and respective WT littermate control mice in three different dosing protocols. In the first protocol, mice received a single 50 µg/kg intra-peritoneal (IP) dose of DT on each of 5 consecutive days and culled 8 days after the first injection. In the second protocol, mice were culled before or 2, 3, 4, 5 and 7 days after receiving a single 50 µg/kg IP dose of DT, and in the third group (Day 0), mice did not receive DT treatment and served as baseline controls. Some mice were DT treated and calcein (5 mg/kg) (Sigma) double-labelled.

### 4.3. Calcium and Phosphorus Measurements

Calcium and phosphorus levels were measured in the serum of 4 groups of 10-week-old male mice (Groups 1 and 2 contained WT, and Group 3 and 4 were Tg mice). WT mice (Groups 1 and 2) were treated by intraperitoneal administration with either: (1) phosphate-buffered saline (PBS) (untreated WT (*n* = 3)) or (2) 50 µg/kg DT (*n* = 6) (ref D0564, Sigma, treated WT); and Tg mice (Groups 3 and 4): (3) PBS treated Tg (*n* = 4) or 50 µg/kg DT (*n* = 3) treated. Seven days after the intraperitoneal injection, blood was collected from the eye and centrifuged. Serum was processed for the measurement of calcium and phosphorus using Architect C8000 automat (Abbott, Lake Forest, IL, USA). Data are expressed in mmol/L.

### 4.4. Health Evaluation

To assess the health status after DT treatment, two groups of mice, Tg (*n* = 4) and WT (*n* = 5), were monitored twice each day for 7 days. The monitoring criteria were body surface temperature, body weight, gait, coat condition, skin tenting for dehydration, hunching, activity, breathing rate and general body condition. Mice were scored blind for each one of these criteria from 1–5, except for body weight and temperature.

### 4.5. Histological Analysis

Tissues were fixed in 4% formaldehyde (from paraformaldehyde Alfa Aesar Inc., Ward Hill, MA, USA) for 48 h at 4 °C prior to routine processing into paraffin and production of slides sectioned at 4 μm stained with haematoxylin and eosin. Stained slides were analysed by a pathologist. Total animals examined were: control, WT (*n* = 4) and Tg (*n* = 4); 3 days post-DT: WT (*n* = 3) and Tg (*n* = 4); 7 days post-DT: WT (*n* = 3) and Tg (*n* = 4). In experiments in which mice were injected with calcein labels, tibiae were processed for histomorphometric analysis. Briefly, tibiae were methyl methacrylate embedded and block faces prepared to provide tibia cross-sections at mid-shaft. Blocks were studied using a Leica SP2 confocal microscope, 488-nm laser line for excitation of >510-nm green-yellow fluorescence of calcein labels, using 10/0.40, 20/0.75 and 40/1.25 objectives. Inter-label distances (for mineral apposition rate) were measured at 10–20 locations around the circumference at both endosteal and periosteal surfaces in tibia mid-shaft sections using ImageJ (Rasband, W.S., ImageJ, U.S. National Institutes of Health, Bethesda, MD, USA) [48]. For routine histological analysis, bones were decalcified with 14% EDTA and processed normally for standard histology, and empty lacunae (as a measure of osteocytes ablation) were counted using ImageJ. For HDTR immunohistochemical staining, tissues were demasked with 0.1% trypsin in PBS at 37 °C for 30 min. Sections were then washed 3 times, each for 5 min with PBS and treated with 0.3% H_2_O_2_ for 30 min at room temperature to block endogenous peroxidase. Tissues were then blocked with 10% rabbit serum for 30 min, and the primary HDTR antibody was added at a 1:100 dilution overnight at 4 °C in a humidified chamber; incubation with PBS lacking primary antibody was used as a negative control. Sections were then washed, and horseradish peroxidase (HRP)-conjugated rabbit anti-goat secondary antibody was added for one hour at room temperature. HRP activity was detected by the DAB (3,3’-diaminobenzidine) method reacted for 5 min. Tissue sections were then counter-stained with methyl green solution.

### 4.6. TUNEL Staining

Apoptosis was evaluated 7 days following single DT injection using the Dead-End TUNEL kit (Promega, Madison, WI, USA) in six 1 mm regions of cortical bone situated 5 mm below the growth plate using ImageJ software (National Institute of Health USA). Briefly, paraffin sections were de-waxed, and re-hydrated then slides were incubated in 0.85% NaCl for 5 min at room temperature and washed 3 times in PBS. Sections were post-fixed in 4% PFA (paraformaldehyde) for 15 min at room then permeabilised by treatment with 20 μg/mL proteinase K for 15 min at room temperature. The tissue was equilibrated in equilibration buffer at room temperature for 10 min before being labelled with terminal deoxynucleotidyl transferase (TdT) reaction mix for 1 h at 37 °C. The reaction was stopped by immersing the slides into 2× SSC buffer for 15 min. The sections were then washed in PBS, blocked in 0.3% H_2_O_2_ for 5 min at room temperature, washed in PBS and incubated in streptavidin HRP antibody for 30 min at room temperature. For positive control tibial sections pre-treated with DNAse I for 10 min and for the negative control, no TdT was performed. Staining was visualized by incubation in DAB solution at room temperature for 5–10 min, and analysis were performed on cortical bone situated 5 mm below the growth plate.

### 4.7. TRAP Staining

Osteoclasts in bone tissue sections were identified using a reaction protocol for tartrate-resistant acid phosphatase (TRAP), which is present within the osteoclast cytoplasm. Sections were de-waxed, re-hydrated and incubated in pre-warmed sodium-tartrate buffer at 37 °C for 5 min, then incubated in Solution A for 30 min at 37 °C, followed by 15 min in Solution B. Finally, sections were rinsed in tap water and counterstained with haematoxylin. The numbers of TRAP-positive osteoclasts were quantified in a 5 mm-long segment of the endosteal surface of cortical bone, 5 mm below the growth plate.

### 4.8. Total RNA Isolation and PCR

For isolation from bones, the right and left tibiae were carefully dissected and all their surrounding muscle removed, leaving the periosteum intact. The cartilaginous ends of the bones were removed and the remaining shaft spun at 5000 rpm for 2 min (Eppendorf Centrifuge, Centrifuge 5804 R, Hamburg, Germany) to remove marrow before being snap-frozen in liquid nitrogen. All other tissues were snap frozen in liquid nitrogen. Frozen tissues were pulverized under liquid nitrogen using a mortar and pestle and lysed in Qiazol lysis reagent (Qiagen, Crawley, West Sussex, UK). Total RNA was purified and DNase-treated using the Direct-zol™ RNA MiniPrep Kit (Zymo Research; Irvine, CA, USA). Prior to cDNA synthesis, the quantity and the integrity of the purified RNA were assessed. cDNA was synthesized from 150 ng of total RNA using a high capacity cDNA reverse transcription kit (Applied Biosystems, Foster City, CA, USA). For PCR reaction, the probes used were: sense ACCCTCCCACTGTATCCACG and antisense ATGAGAAGCCCCACGATGAC for *HDTR*; and sense CGGCTGGTGGACTCTCTAAG and antisense CGGGGTCGTCGCTCTGCATC for *Dmp1* [49]; the reaction was run on a 60 °C annealing temperature. PCR amplicons were sequenced and restriction digested.

### 4.9. Quantitative PCR

For qPCR, qPCRBIO SyGreen 1-Step Lo-ROX (PCR Biosystems, London, UK) was used to perform qPCR with the absolute quantification method. Standards for each gene of interest were generated, and the copy number of genes of interest was quantified using the Bio-Rad CFX manager software; then, the results were normalised to GAPDH, which showed no significant difference when comparing each selected Tg tissue to its WT counterpart. qPCR reaction primers with amplification efficiency between 90% and 110% and an R2 standard curve value between 0.99 and 1.00 were considered acceptable. Primers’ specificity was demonstrated with a single peak of the melting curve with a specific temperature. The reaction buffer contained, 5 μL of 2× qPCRBIO SyGreen 1-Step Mix, 0.4 μL forward primer, 0.4 μL reverser primer, 1.0 μL 20× RTase, 30 ng of RNA and H_2_O up to 10 μL. Running conditions were one cycle of 55 °C for 10 min, 95 °C for 2 min for reverse transcription, 40 cycles of 95 °C for 5 s for denaturation and 60 °C for 30 s for annealing/extension. *HDTR* sense primer sequence: CTTATATACCTATGACCACACAACC, and antisense: CACGATGACCAGCAGACAG were used.

### 4.10. Western Blotting

Total protein was extracted using RIPA lysis buffer (Thermo Scientific, Loughborough, UK) from a wide range of liquid nitrogen snap-frozen tissues (heart, spleen, muscle, lung, kidney and liver) from Tg mice before and 7 days after DT treatment (*n* = 4 for each), and samples were not pooled. Protein concentrations for each sample were determined by the Pierce BCA Protein Assay Kit BCA assay (Fisher, Paisley, UK). Forty micrograms of protein were size-fractionated using SDS-PAGE and electrotransferred onto polyvinylidene difluoride (PVDF) membranes (Schliecher and Schuell, Dassel, Germany). Membranes were blocked for 1 h in 0.2% (*w*/*v*) I-block (Topix, Bedford, MA, USA) before being incubated with primary anti-mouse phospho-eEF-2 rabbit mAb (1:1000; Abcam, Cambridge, UK) and secondary anti rabbit IgG (1:2000; Cell signalling Technologies, Danvers, MA, USA) antibodies. Proteins were visualized using the enhanced chemiluminescence detection system (ECL) (Fisher, Paisley, UK). After visualisation, the intensity of total EF-2 and pEF-2 proteins bands form each treated and vehicle Tg mice was quantified using ImageJ. For the results, representative blots form each sample were presented, and quantification was presented as the percent of change of pEF-2 against total EF-2 from all samples. 

### 4.11. Statistical Analysis

Data were checked for a normal distribution using the D’Agostino and Pearson omnibus normality test. To compare between two groups, Student’s unpaired *t*-test was used. In comparisons between more than two groups, one-way analysis of variances (ANOVA) was used with a Tukey post hoc comparison. All statistical analyses were performed using GraphPad Prism 6 (GraphPad Software, Inc., San Diego, CA, USA), and the results are expressed as the mean ± the standard error of the mean (SEM). *p* < 0.05 was considered to be significant.

## 5. Conclusions

Overall, these data demonstrate that there are confounding effects in using this model to identify osteocyte-specific functions. The method of combining selective exposure to DT with tissue restricted expression of *HDTR* initially appeared to represent a specific method for acute ablation of osteocytes, but our data question this selectivity. This system proved more complex than originally reported, and our attempts to use this model have instead highlighted system-wide effects and significant deterioration of health status. Indeed, the multitude of changes after DT treatment rendered it impossible to effectively use this model to study osteocyte function in vivo. In addition to highlighting the necessity for extensive characterisation of transgenic mouse models, our findings also emphasise that future studies involving *HDTR* Tg mice take into account the complex systemic changes that occur when drawing conclusions from experimental data. For these reasons, we also urge that alternative models for achieving osteocyte selective ablation are sought.
